# Usability and cultural adaptation of a text message-based tobacco cessation intervention for people living with HIV in Uganda and Zambia

**DOI:** 10.1186/s13722-025-00580-z

**Published:** 2025-07-20

**Authors:** Heather Wipfli, Jim Arinaitwe, Fastone Goma, Lynn Atuyambe, David Guwatudde, Masauso Moses Phiri, Elizeus Rutebemberwa, Fred Wabwire-Mangen, Richard Zulu, Cosmas Zyambo, Kyra Guy, Ronald Kusolo, Musawa Mukupa, Ezekiel Musasizi, Joan S. Tucker

**Affiliations:** 1 Department of Population and Public Health Sciences, Keck School of Medicine, University of Southern California, 2001 N. Soto Street, Los Angeles, CA 90033 USA; 2 School of Public Health, Centre for Tobacco Control in Africa, Makerere University, Kampala, Uganda; 3 School of Medicine, Centre For Primary Care Research, University of Zambia, Lusaka, Zambia; 4 Department of Community Health and Behavioural Sciences, School of Public Health, Makerere University, Kampala, Uganda; 5 Department of Epidemiology and Biostatistics, Makerere University School of Public Health, Kampala, Uganda; 6 Department of Pathology and Microbiology, School of Medicine, University of Zambia, Lusaka, Zambia; 7 Department of Health Policy, Planning, and Management, School of Public Health, Makerere University, Kampala, Uganda; 8 Department of Community and Family Medicine, School of Public Health, University of Zambia, Lusaka, Zambia; 9 RAND Corporation, Santa Monica, CA USA

**Keywords:** Tobacco, Cessation, HIV, Text messaging, Intervention, Sub-Saharan Africa

## Abstract

**Background:**

Text messaging-based interventions (TMIs) have demonstrated effectiveness in reducing tobacco use in many populations. However, such interventions have not been tailored to meet the complex medical and psychosocial factors confronting people living with HIV (PLWH) in sub-Saharan Africa (SSA). We describe the process of adapting the SmokefreeTXT message library so that it is applicable to all forms of tobacco use, addresses issues specifically facing PLWH who use tobacco, and is culturally appropriate for use in Uganda and Zambia.

**Methods/design:**

Participants were PLWH who currently used tobacco and health services workers recruited from HIV clinics in two regions of Uganda and two regions of Zambia. Eight focus groups (*N* = 48) were conducted with PLWH tobacco users and four focus groups (*N* = 28) were conducted with healthcare providers to adapt the TMI content for the cultural context and HIV status. A subsample of PLWH focus group participants (*N* = 14) provided feedback on the adapted TMI after using it for three weeks on their own phone. Focus group transcripts were analyzed for key themes based on the moderator guides using Dedoose software™. Means and percentages were calculated for survey data to assess the TMI’s acceptability and feasibility.

**Results:**

Focus group feedback on facilitators and barriers to quitting tobacco, as well as strengths and limitations of the TMI-based intervention approach, were used to finalize the adapted TMI’s content and delivery for usability testing. PLWH identified multiple barriers to quitting tobacco including addiction, lack of support and education, and community perceptions. Health service workers highlighted the need for community-level interventions, improved provider knowledge on tobacco cessation, and tailored support strategies. Usability testing participants rated the TMI as helpful and relevant, emphasizing the interactive features as supportive and beneficial. Further, they reported few problems using it over three weeks, except for difficulty keeping their phone charged.

**Conclusions:**

Results suggest that an adapted version of SmokefreeTXT is a feasible and acceptable option for PLWH in Uganda and Zambia who are interested in quitting tobacco use.

*Trial registration*: ClinicalTrials.gov Identifier NCT05487807. Registered August 4, 2022, https://clinicaltrials.gov/ct2/show/record/NCT05487807

## Background

Despite advancing therapies and treatments, Acquired Immunodeficiency Syndrome (AIDS)-related morbidity and mortality among people living with HIV (PLWH) continue to pose significant public health challenges in sub-Saharan Africa (SSA) [1]. In Uganda and Zambia, for example, the HIV prevalence rate among 15–49 year olds is 5.1% and 10.8%, respectively [2]. As is the case elsewhere, tobacco use in SSA is more common among PLWH than non-PLWH; for example, studies have found that PLWH in Uganda are twice as likely to use tobacco than the local population (20% vs 10%) [3], and the prevalence of tobacco use in Zambia is higher among PLWH than non-PLWH for both women (4.4% vs. 2.4%) and men (27.8% vs. 18.7%) [4]. Tobacco use among PLWH is especially concerning in that it contributes substantially to the HIV burden [5]. Research on tobacco cessation in PLWH presents unique challenges, including higher rates of depressive symptoms and mental health concerns, greater use of alcohol and other substances, lack of knowledge on the impacts of tobacco, lower interest in quitting, and poorer adherence and response to antiretroviral therapy [6–9]. Effective and feasible tobacco cessation interventions are needed for PLWH in SSA, especially in light of the high rate of tuberculosis (which is worsened by smoking) in this population [10]. However, a recent systematic review of tobacco cessation intervention evaluations in SSA that included a control group and reported on tobacco quit rates identified just eight studies—all conducted in South Africa and only one focusing specifically on PLWH (which compared the effects of intensive anti-smoking counseling alone vs. counseling plus nicotine replacement therapy) [11]. As identified by the review, numerous barriers exist to implementing tobacco cessation support in SSA. For example, healthcare providers face barriers to delivering cessation advice including personal smoking behaviors, lack of knowledge and training, limited awareness or motivation, and overburdened clinics [11].

These barriers emphasize the need to integrate sustainable tobacco cessation interventions within existing health systems. With mobile phone ownership steadily growing in SSA [12], automated text messaging interventions (TMI) may be an attractive and scalable treatment strategy in this region. The promise of this approach is bolstered by research studies conducted in Uganda and Zambia that have found mobile phone-based interventions to be feasible, inexpensive, and viewed favorably by patients and providers [13–15]—albeit none have specifically addressed tobacco cessation among PLWH. However, more generally, there is growing evidence of the effectiveness of TMIs in helping adults quit tobacco [16, 17]. For example, a Cochrane Systematic Review concluded that automated TMIs result in greater quit rates than minimal smoking cessation support and that the effectiveness of other smoking cessation interventions can be enhanced by adding a TMI component [18]. Recognizing the potential of mobile phone-based interventions to address barriers to health care in low and middle-income countries (LMICs) [19, 20], WHO and the International Telecommunications Union launched *Be He@lthy, Be Mobile* in 2012, which helps countries build, scale, and sustain digital health programs addressing non-communicable diseases and their risk factors, including a TMI program focused on tobacco cessation [21]. Using the *Be He@lthy, Be Mobile* framework, we are adapting and evaluating an existing evidence-based TMI for PLWH in Uganda and Zambia who are interested in quitting tobacco. As described in more detail elsewhere [22], the evaluation involves a four-arm randomized controlled trial (RCT) comparing the relative effectiveness of usual care, the adapted tobacco cessation TMI (entitled *Quit4Life* +), nicotine replacement, and the combination of *Quit4Life* + and nicotine replacement on tobacco cessation over six months.

This paper presents results from the formative phase of this project, focusing on the cultural adaptation of the TMI intervention. Cultural adaptation involves modifying evidence-based treatments to incorporate the unique characteristics of the target population, such as local language, social norms, and spiritual beliefs [23]. This critical process has been shown to enhance sustainability and improve users’ attitudes and acceptability of interventions, particularly in low- and middle-income countries [24, 25]. Several studies have proposed models for the successful cultural adaptation of technology-supported interventions, emphasizing best practices such as integrating feedback from local communities through focus groups and key informant interviews and pilot testing materials to ensure relevance and effectiveness [26, 27]. Guided by these models, the formative phase of the QuitforLife + trial included focus groups and usability testing of text messages. Specifically, we conducted focus groups with PLWH who use tobacco, as well as health services workers (HSW) who provide services to these patients in local HIV clinics. The goal of the focus groups was to explore attitudes, beliefs, and perceived barriers to tobacco cessation, as well as preferences for program delivery (e.g., number and timing of texts), to inform the adaptation of the existing TMI library. For example, in the case of developing this intervention in the context of PLWH in Uganda and Zambia, the adapted library needed to be relevant to both combustible and noncombustible forms of tobacco use (the latter being more common in SSA), the unique issues related to tobacco cessation among PLWH, and the cultural climate of tobacco use and cessation in SSA. Second, we describe results from the usability testing subsequently conducted with a subset of the PLWH focus group participants. Following focus groups, participants were asked to use the adapted TMI for three weeks and provide feedback on its acceptability, feasibility, and appropriateness through established usability testing methods employed previously by the study team [28]. Final refinements to *Quit4Life* + were made based on usability testing results before commencing the RCT.

## Methods/design

### Participants and recruitment

#### PLWH focus groups

Participants were initially recruited for focus groups at four clinics serving PLWH, two in Uganda (Arua, Moroto) and two in Zambia (Mongu, Chipata). Eligibility criteria were: (a) being age 18 or older; (b) being HIV positive and receiving ART services at one of the participating clinics at the time of the recruitment visit; and (c) using any type of tobacco in the past 30 days. At each clinic, an expert client was identified and enlisted to help contact eligible focus group participants. Due to the stigma of tobacco use by women in the geographic regions of the study, separate focus groups were conducted at each clinic for men (26 participants total; 6–8 per group) and women (22 participants total; 2–8 per group), resulting in a total of eight focus groups.

#### HSW focus groups

These participants were recruited across the participating health facilities for separate focus groups. One focus group was conducted at each of the four clinics serving PLWH just described (6–9 participants per group), resulting in four focus groups involving a total of 28 HSWs. Eligibility criteria for focus group participation were: (a) being age 18 or older; (b) working in a health services capacity at the clinic; and (c) having some knowledge of HIV and tobacco use.

#### PLWH usability testing

At a later point in time, after SmokefreeTXT had been adapted based on focus group feedback, participants were re-contacted to assess their interest in participating in the three-week usability testing of the short message service (SMS) program. To be eligible for this phase of the study, they needed to have their own phone and agree to be sent text messages. Of the 48 focus group participants, 14 (9 men, 5 women; mean age = 45.6) participated in the usability testing and returned for follow-up data collection three weeks later.

All participants provided informed consent and were compensated 10 USD for focus group participation, usability testing, and survey completion. All aspects of the study were approved by the institution’s internal review boards.

### Focus group discussion

Two facilitators were present at each focus group: one facilitator moderated the discussion, and another assisted with the note-taking, materials, and logistics. Focus groups were conducted in the local language. The focus group discussions were audio-recorded with permission from the participants. These were then directly transcribed into English because the facilitators spoke fluently the local language as well as English. For quality control, the two transcribers read each other's transcript for both content and completeness before sending them to the investigators who also read the transcripts and made comments. These comments were sent back to the transcribers to re-listen to the audios for content and completeness of the data.

#### PLWH focus groups

At the beginning of the focus group, participants were told that we would like their feedback on a SMS program to help PLWH quit tobacco: “The goal of this program is to help patients like you quit tobacco and to stay tobacco-free. Patients who use the program will be asked to set a quit date. Once they sign up for the program, they will be provided with a cell phone and then will start receiving text messages that will provide ongoing support throughout the quitting process. Messages will be sent in the days leading up to their quit date, on the actual day they quit, and then for 3 months after their quit date.” Participants were then told that we would be sharing with them some examples of text messages about quitting tobacco so they could get an idea of what the program would look like. They were shown text messages that would be sent prior to their quit date (to prepare for quitting), on their quit date, and after their quit date (to provide continued support for quitting)—a total of 23 text messages were shared (see Table 1). Using a focus group guide, participants were systematically asked to provide feedback on the content of each group of messages (e.g., pre-quit day, quit day, post-quit day), what they thought of the interactive features of the texts, and other messages or links to other information that they thought would be useful during the quitting process.

Participants were informed that the program would be designed to send text messages after quit day to provide ongoing support for staying tobacco-free, with the texts focusing on topics such as: (a) dealing with cravings; (b) staying motivated to quit; (c) getting social support for quitting; and (d) beneficial outcomes associated with quitting. For each topic, participants were asked to identify specific barriers to quitting that the program should address. Finally, participants were asked five additional questions: (a) what was missing from the list of topics in terms of issues that the SMS program should address to help tobacco users like them quit; (b) whether certain issues were more important than others and should be focused on more with the text messages; (c) if they were trying to quit tobacco and found themselves in a situation where they were really tempted to use it, what would be most helpful in terms of getting them through the situation and staying tobacco-free; (d) what was missing from the SMS program that they thought would be useful to help tobacco users like themselves quit; and (e) what other suggestions did they have for helping us improve this program.

#### HSW focus groups

The discussion first focused on identifying major barriers that HSWs might encounter in helping PLWH quit using tobacco, barriers that would be specific to using a SMS program to help PLWH quit tobacco, and identifying strategies for addressing these barriers. Participants were also asked to identify other issues that the research team should be aware of in helping HSWs to implement a tobacco cessation program for PLWH. Participants were then asked to identify the most critical elements in tobacco cessation services for PLWH, the most effective tobacco cessation program to use with PLWH, the core or critical elements of this program, and what changes or adaptations would need to be made to an existing program to make it as effective as possible for PLWH. Finally, they were asked for suggestions to encourage PLWH to participate in a tobacco cessation program.

### Usability testing

As stated earlier, PLWH who participated in a focus group discussion were recontacted and, if interested, invited to participate in the usability testing of the adapted SMS program. Specifically, they were asked to set a quit date and to use the adapted SMS program on their own phone for three weeks. Participants received pre-quit day messages for two weeks and quit-/post-quit day messages for one week. They then returned to the clinic to complete a post-usability testing survey.

### Survey measures

A short paper–pencil survey was completed by all focus group and usability testing participants. The surveys were administered in the local language of the region where the focus group was conducted. Participants who could complete the survey did it on their own, whereas those who could not read nor write the local language had the survey items read to them.

#### PLWH focus group survey

Participants completed the survey immediately after the focus group discussion. Demographic information included self-reported sex (male, female) and age. Tobacco use variables included time to first tobacco use of the day (an indicator of nicotine dependence; 1 = *within 5 min* to 4 = *after 60 min*) [29], and the number of times they quit tobacco for at least 24 h in the past year. Preferences for TMI delivery were assessed by asking participants how many messages they would want to receive each day if they were using a SMS program to help them quit tobacco use, with reference to five periods: one day before quit day; quit day; one day after quit day; one week after quit day; and one month after quit day (response options ranged from 1 per day to 10 or more per day). They were also asked what time of day they would want to receive these SMS messages (they could choose more than one): first thing in the morning; later in the morning; in the afternoon; and in the evening. Feasibility was assessed by asking participants about the perceived likelihood of experiencing five types of phone-related problems if they were using an SMS program: trouble keeping their phone battery charged; losing their phone; having their phone stolen; being concerned about people seeing them with a new phone; and being concerned about people seeing messages about quitting tobacco use on their phone (1 = *very unlikely* to 4 = *very likely*). Acceptability and relevance were assessed by asking participants how helpful they would find the SMS program that was discussed if they were trying to quit tobacco, how likely it is that they would use a SMS program like the one discussed if they were trying to quit tobacco, and the relevance of the text messages presented to them to their own situation. Finally, they were asked to list up to 3 things we should change or improve about the SMS program to make it more appealing and relevant for PLWH who want to quit using tobacco.

#### HSW focus group survey

Participants completed the survey immediately prior to the focus group discussion. Demographic information included self-reported sex (male, female) and age. Professional background included their position (job title) and length of employment within their organization, whether they had training or certification in providing HIV/AIDS services, whether they had been involved in delivering a tobacco cessation program, number of years they had worked in HIV/AIDS services, and whether they had worked specifically on issues related to tobacco use among PLWH.

#### PLWH usability testing survey

Usability testing participants completed a survey prior to the usability testing to assess demographics and tobacco use, and another survey after the usability testing to assess their preferences for text message delivery, and feasibility and acceptability of the SMS program based on their experiences using it. Items assessing demographics, tobacco use, and preferences for TMI delivery were the same as just described for the PLWH focus group survey. Feasibility was assessed by asking participants whether or not they had experienced five types of phone-related problems while using the SMS program: trouble keeping their phone battery charged; losing their phone; having their phone stolen; people saw them with a new phone and asked about it in a way that made them uncomfortable; and people could see text messages about quitting tobacco on their phone and it made them uncomfortable. Acceptability and relevance were assessed by asking how helpful the text message program was in helping them quit tobacco, as well as how helpful the texts were when they were experiencing a craving, having trouble staying quit, and keeping motivated to quit. Usability testing participants were also asked how likely it is that they would use this type of program if they were trying to quit tobacco in the future, and how relevant the text messages were to them. All ratings were on a scale from 1 = *not at all* to 5 = *extremely*.

### Data analysis

#### Quantitative survey data

Means (standard deviations) and percentages were calculated for the quantitative survey items.

#### PLWH qualitative focus group data

Transcripts of the PLWH focus group discussions were reviewed by study personnel. These transcripts were then imported into Dedoose™. One member of the research team developed the first draft of the codebook, based initially on the focus group moderator guide, but expanded at the early stages of coding when additional relevant topics emerged. Specifically, themes focused on each topic area that was asked about during the discussion (e.g., dealing with cravings, motivation to quit, getting social support, benefits of quitting). Themes were also identified pertaining to barriers to quitting, the use of mobile phones to deliver the intervention, tobacco control interventions at the community level, and tobacco source and income. Finally, specific program suggestions had its own code in order to easily flag feedback for what could be added or changed related to the content or delivery of the program. Three researchers independently coded excerpts from one transcript, met to discuss the codes and resolve any coding discrepancies, and then finalized the codebook (e.g., refining definitions and inclusion/exclusion criteria for coding excerpts into specific themes). For each of the remaining seven transcripts, two coders independently coded the transcript, then met to discuss the codes and resolve any coding discrepancies, bringing all transcripts into 100% agreement.

#### HSW qualitative focus group data

Given the highly structured format of the HSW focus group moderator guide, we have listed responses under one of three main lines of inquiry from the moderator guide: (a) barriers that health service workers might encounter in helping PLWH quit using tobacco and strategies for addressing these barriers; (b) using SMS as a program to help PLWH quit tobacco and strategies for addressing SMS-related barriers; and (c) critical elements in tobacco cessation services for PLWH. Note that the HSW moderator guide included additional questions asked about the most effective tobacco cessation program to use with PLWH, the core or critical elements of this program, what changes or adaptations would need to be made to an existing program to make it as effective as possible for PLWH, and how to encourage PLWH to participate in a tobacco cessation program. We classified responses to these questions into one of the three main themes.

## Results

### Participant demographics

#### PLWH focus group demographics

The age range of focus group participants was 19–78 years (mean = 41). In terms of tobacco use, 61.7% reported first use of tobacco within 5 min of waking and having quit for at least 24 h an average of 2.06 times (*SD* = 3.83) in the past year.

#### HSW demographics

Of the 28 focus group participants, 61% (*n* = 17) were female. Ages ranged from 26 to 50 years (mean = 34) Their professional positions varied and included nurses or midwives (*n* = 6), HIV counselors (*n* = 5), clinical officers (*n* = 4), in-charge at ART clinic (*n* = 3), medical doctors (*n* = 2), med lab assistant or technician (*n* = 2), and assorted other positions (*n* = 6). They had been with their organization for 1–25 years (mean = 6) and had been working in HIV/AIDS services for 2–21 years (mean = 5.5). Nearly all (96%; *n* = 27) had received training or certification in providing HIV/AIDS services, 14% (*n* = 4) had been involved in delivering a tobacco cessation program, and 11% (*n* = 3) had worked specifically on issues related to tobacco use among PLWH.

### PLWH focus group survey results

#### Preferences for TMI delivery

As shown in Table 1, on average, focus group participants wanted to receive 4 to 6 text messages per day. The number varied somewhat depending on the stage of the quit attempt. For example, focus group participants wanted to receive more text messages on Quit Day than on subsequent days. In terms of when to receive texts (first thing in the morning, later in the morning, afternoon, or evening), participants could choose multiple responses. Focus group participants had a clear preference for receiving messages first thing in the morning.

**Table 1:** PLWH responses to survey items assessing SMS program acceptability

Item	Focus Group (*N* = 48)	Usability Testing (*N* = 14)
*Program helpfulness and relevance (M, SD)*		
Likelihood of using program in the future ^a^	4.6 (0.6)	4.6 (0.5)
Relevance of the text messages ^a^	4.5 (0.9)	4.7 (0.5)
Helpfulness of program in helping you quit ^a^	4.8 (0.4)	4.9 (0.4)
Helpfulness when experiencing a craving ^a^	n/a	4.8 (0.4)
Helpfulness when having trouble staying quit ^a^	n/a	4.7 (0.4)
Helpfulness in keeping you motivated to quit ^a^	n/a	4.8 (0.5)
*If using a SMS program for 3 months, likelihood of the following things happening (M, SD)*		
Trouble keeping phone battery charged ^b^	2.2 (1.3)	n/a
Lose the phone ^b^	1.1 (0.3)	n/a
Phone would be stolen ^b^	1.2 (0.5)	n/a
Concern about being seen with new phone ^b^	1.7 (1.1)	n/a
Concern about people seeing text messages about quitting tobacco on phone ^b^	1.8 (1.2)	n/a
*The following things happened when you were using the SMS program (N, %)*		
Trouble keeping phone battery charged	n/a	5 (36%)
Lost phone	n/a	3 (21%)
Phone was stolen	n/a	3 (21%)
People saw me with new phone and asked about it in a way that made me uncomfortable	n/a	2 (14%)
People could see text messages about quitting tobacco on phone and it made me uncomfortable	n/a	2 (14%)
*Preferred number of texts per day (M, SD)*		
On the day before your quit day	5.1 (3.5)	4.2 (2.3)
On your quit day	5.5 (3.5)	4.1 (2.3)
On the day after your quit day	4 (2.8)	4.6 (2.6)
One week after your quit day	4.5 (2.9)	4.6 (2.9)
One month after your quit day	4.9 (3.2)	5.7 (3.4)
*Preferred times of day to receive texts (N, %)*^*c*^		
First thing in the morning	17 (35%)	6 (43%)
Later in the morning	6 (13%)	6 (43%)
In the afternoon	4 (8%)	6 (43%)
In the evening	4 (8%)	6 (43%)

^a^ Scale: 1 = *not at all* 2 = *somewhat*, 3 = *moderately*, 4 = *very*, 5 = *extremely*. ^b^ Scale: 1 = *very unlikely*, 2 = *somewhat unlikely*, 3 = *somewhat likely*, 4 = *very likely.* ^c^ Participants could choose more than one option

#### Feasibility and acceptability of TMI

As shown in Table 1, focus group participants rated the SMS program as being “very” to “extremely” helpful if they were trying to quit tobacco and relevant in terms of text message content. Further, they indicated that it was “very” to “extremely” likely that they would use a program like this one if they were trying to quit tobacco. In terms of potential problems if they were using the SMS program for 3 months, the focus group participants thought that it was “somewhat unlikely” that they would have trouble keeping their phone charged, “very unlikely” that their phone would be lost or stolen, and between “somewhat” and “very” unlikely that they would be concerned about being seen with a new phone or about others seeing text messages about quitting tobacco on their phone.

Table 2 shows responses to the write-in item asking PLWH focus group participants to list up to 3 things we should change or improve about the SMS program to make it more appealing and relevant for PLWH who want to quit tobacco. Most suggestions focused on the delivery of the SMS content, such as using a language they understand, audio recording the messages for those who cannot read, providing solar panels to charge phones, keeping messages short and brief, using pictures which show the dangers of tobacco use, and providing an alternative way to access the messages. However, some participants also had suggestions for the content of the messages, such as talking about the health risks of tobacco use. Several of the suggestions referred to adjunct components that could be added to the SMS program such as providing social and financial support, having frequent meetings with participants, and promoting tobacco cessation through radio talk shows. Finally, there was a suggestion to involve all PLWH in the program.

**Table 2:** Number of PLWH focus group participants who mentioned a particular suggestion for refining Quit4Life + to be more appealing/relevant (*N* = 48)

Suggestion	
*Literacy and alternatives to SMS delivery*	
Messages should be in language we understand	4
Audio record messages because I cannot read	2
Use pictures which show dangers of tobacco use	3
Sensitization through drama kits	1
Radio talk shows	1
Provide alternative way to access messages	1
Flyers on tobacco messages	2
*Resources*	
Provide phones, chargers, money for airtime, etc	9
Provide financial support/food relief	5
*Message content and delivery*	
Messages should be brief	1
Send messages consistently	5
Talk about the dangers of tobacco to our health	6
*Have regular meetings/follow-up with us*	6
*Engage others in the program*	
Inform health workers	1
Inform local leaders	2
Engage people supported by Ministry of Health/Donors	2
Mobile team to sensitize the hazards of smoking	4
*Issues regarding PLWH status*	
Involve all PLWH in this program	1
Target only PLWH	1
Empower PLWH	1
Treat PLWH well so that they will comply	1

Table shows the number of participants who mentioned a particular suggestion. Participants could list up to three suggestions

### PLWH focus group qualitative results

Four hundred and forty-five (445) individual excerpts were identified and coded into themes within nine topic areas (see Table 3): program suggestions (excerpts that specifically suggested things that could be added or changed related to the content or delivery of the program); barriers to quitting; benefits of quitting; dealing with cravings; getting or staying motivated; social support; phone-based delivery; tobacco control interventions at the community level; and tobacco source and income. Related subthemes were applied under several of these topic areas, resulting in 23 distinct codes. There were 774 code applications on the 445 excerpts, meaning that many excerpts were given multiple codes.

**Table 3:** PLWH focus group themes, description of themes, and example quotes

Theme	Description	Subthemes (if applicable) and Example Quotes
Barriers to quitting	Statements about factors that made quitting tobacco difficult	**Addictive nature of tobacco:** *“One of the barriers to quitting tobacco is an addiction. Most of us are very addicted to using tobacco to the extent that if we don’t use it (tobacco), we don’t normally feel very normal and therefore, it makes it hard for us to be convinced that tobacco used is harmful to our health (1).”* **Use of other substances:** *When you drink alcohol there is an immediate need for tobacco so that the whole thing looks complete (2).”* **Beneficial effects of tobacco on hunger or alertness:** *People who take tobacco always say that they are always hungry and they claim that when one sniffs tobacco, the hunger is quenched. So that’s what makes it hard for them to quit tobacco (3).”* **Community perceptions and practices:** *“For me I think one of the barriers could be people do not perceive tobacco use as something harmful since this is something done within their community; so tobacco use is never regarded as something bad. Such is the support that we really lack in our community (4).”* **HIV status:** *Most of us use tobacco because we feel there is nothing much we can do. So we use it in order to keep ourselves free from that trauma. Therefore, I find this poor mindset as one of the major barriers to quit using tobacco (5).”* **Knowledge and attitudes about quitting**—this subtheme was further split into: ● **Healthcare worker knowledge and attitudes about quitting:** *“I think there is also an issue of knowledge gap; why I am saying this is because most trusted people who are closest to us are the healthcare workers and yet they also don’t have adequate knowledge about how to deal with tobacco related issues (6).”* ● **Individual knowledge and attitudes about quitting:** “*Maybe some messages that would be informing someone how harmful smoking is to a PLWH so that the person permanently quit; yeah a message of this kind (7).”* **Negative moods and emotions:** *“I think one of the barriers is attitude related; as in most of us are idle. We so much perceive ourselves as if we are the most vulnerable people to the extent that we feel that we cannot do anything to keep ourselves occupied which to me always brings in the craving issue that we just talked of (8).”*
Program suggestions	Statements about program content or delivery that could be added or changed	*“It would also be nice if this program includes some follow up; as in to try and find out whether people are using and really changing as a result of the program because some might end up selling these phones to fulfill other needs. You can also include some audio-recording for those who cannot read or write (9).”*
Phone-based delivery	Statements about the use of phones to deliver tobacco-cessation program	**Compared to in-person care:** *“These texts create an environment similar to that of the health Centre. It is really friendly.” “Generally these texts are all good because they are communicating the benefits of quitting tobacco use to us and such information may never be obtained from anywhere, not even at the health Centre where we get our medication (10).”* **Convenience and cost:** *“You know something that comes direct to your phone gives you plenty of time to do other things as opposed to walking a very long distance from your home to the health facility just to go and see the Doctor. I find this really saving a lot of time.” “The messages have been designed in a way that you do not have to incur any cost meaning you can reply to a message even if you do not have credit on your phone. This is really very nice (11).”* **Interactive features:** *“The program gives you the freedom to control the flow and providing you the means to reply back a message is something I like so much about this program.” “The messages are truly interactive; you have the right to respond to your need as in what challenge you are facing (12).”* **Length of program or messages:** *“Some of the texts are really very long; brief and precise texts would be much better (13).”* **Literacy issues:** “*Include some audio-recording for those who cannot read or write (14).”* **Privacy issues:** *“This is something very exciting about this program; the message coming directly to my phone is very great. No one has to know about what I am doing (15).”* **Tone of text messages:** *“To me these texts are all meaningful and they are very polite; like the text which asks whether you are ready is just like someone seeking your consent to do something which to me is very important (16).”*
Social support	Statements about getting or having support from others for quitting, not wanting to get support from others, lack of support for quitting at either the individual-level or community-level, and providing support to others for quitting. Includes comments about stigma/discrimination	*“I like the second text which talks about you sharing the good news with friends because the moment you inform your friend about the great news, then this friend will be able to keep you motivated to quit (17).”* *“For me I have issues with the last text because I don’t think whether everyone would love to inform someone about what he/she ought to do; if I have decided to quit tobacco, do I really have to inform someone? I don’t think that is right (18).”* *“This is really true because most of us don’t have such friends that encourage us to quit but rather we have friends who encourage us to keep on using tobacco (19).”*
Dealing with cravings during quit attempt	Statements about the importance of focusing on cravings, barriers to dealing with cravings, or suggestions for how to deal with cravings	*“I think the best help would be what I need to do to eliminate the cravings that still do persist in me so that I don’t ever think of using tobacco again (20).”* *“I would suggest that it would be really good to have some examples of messages related to cravings such that we get an understanding of some of the ways to deal with them (21).”*
Getting or staying motivated	Statements about the assessment of motivation to quit, getting motivated, or staying motivated—excludes statements about negative moods and emotions	*“Because most people here do use tobacco; the youth, the elderly, and even some of our women do use tobacco. So who is there to advise who? Therefore, there is no one you can rely on to keep you motivated (22).”* *“I like one that says ‘keep staying strong’ this is because it needs someone to take heart to really get off something that he has been used to doing. I also find this as a motivation to overcome craving that was mentioned earlier (23).”*
Benefits of quitting	Statements about the positive aspects or outcomes associated with quitting tobacco	**Financial:** *“Another useful example of text is the one which talks about you trying to estimate the amount you spend on tobacco products every day in a year, it could be a lot. Like a member said, we don’t have financial education which makes us use whatever monies we have in a reckless manner (24).”* **Health:** *“To my understanding, the second text is quite good. It explains to us how certain disease conditions are attributable to tobacco use and thus we should quit using tobacco to stay free from such diseases (25).”* **Social**: *“I like the second text which says that “Quitting will prevent family members from disease associated with living with a smoker.” This also give one an insight into the social benefits of quitting tobacco use because it states that quitting does not only benefit the health of the smoker but also those closely in contact with them (26).”*
Tobacco control interventions at the community level	Statements about the need for community-level intervention (CLI), existing CLIs in their village or elsewhere, or what a CLI might include	*“I think one of the barriers could be things to do with lack of proper supportive structures within the community that one would receive proper guidance on how to deal with cravings (27)”* *“You should actually add our elders in the system because they are the ones who actually know how the tobacco issue cqme up and they should be in best position to explain how to reduce and eventually leave tobacco use (28).”*
Tobacco source and income	Statements about where they obtained tobacco, as well as about the cultivation of tobacco and income associated with cultivation	*“Sometimes the most reliable source for free tobacco are the elders because they are the biggest consumers of tobacco, and as such always have plenty of it. Because of this, we all have to be friendly to them in order to get tobacco (29).”* *“For us in our community here this tobacco you are talking about is a source of livelihoods to almost all families because they sell this tobacco to the people and get income. So since the program is targeted on counseling tobacco use in the community (30).”*

Bold values indicate subthemes identified within main themes

#### Barriers to quitting (148 excerpts)

This theme included statements about factors that made quitting tobacco difficult. Seven subthemes were coded. **Addictive nature of tobacco use** included excerpts where clients specifically used some variation of the word “addiction” and mostly focused on how addiction to tobacco made it difficult to quit (Table 3, Quote 1). **Use of other substances** focused primarily on participants noting the use of alcohol as a trigger for tobacco use (Table 3, Quote 2). **Beneficial effects of tobacco on hunger and alertness** included a small number of excerpts referring to tobacco use as a way to curb hunger or to increase/decrease alertness (e.g., help you sleep; help you wake up) (Table 3, Quote 3). Participants discussed many **community perceptions and practices,** primarily focusing on tobacco use being normative and socially acceptable in the community (Table 3, Quote 4). There were a few mentions of HIV-related stigma and isolation in the community, as well as lack of community support for quitting. One participant mentioned the importance of delivering the program countrywide so that everyone receives information about quitting tobacco. **HIV status** focused on how HIV affects one’s ability to quit tobacco; for example, quitting is not a priority because they are already in poor health or, conversely, quitting is important because they need to prioritize their health (Table 3, Quote 5). Several participants mentioned stress, idleness, stigma and isolation, and lack of support as a person living with HIV were barriers to quitting (note that these were likely also coded under “Negative moods and emotions”). **Knowledge and attitudes about quitting** included excerpts focusing on knowledge and attitudes related to tobacco use/quitting, harms from use, or financial literacy that make it more difficult to quit. Two child codes were applied, one for excerpts referring to their own lack of knowledge and need for accurate information about tobacco and how to quit, and the other for excerpts referring to a knowledge gap on these issues among healthcare providers (Table 3, Quote 6,7). **Negative moods and emotions** included excerpts describing how quitting tobacco is a challenge due to feelings of depression and hopelessness, stress and anxiety, boredom and idleness, and stigma and isolation (Table 3, Quote 8).

#### Program suggestions (128 excerpts)

This theme included statements suggesting content for the text messages (including specific recommendations for dealing with cravings), delivery of the tobacco cessation program, other components that could be added to Quit4Life +, and other considerations in developing the program (Table 3, Quote 9).

#### Phone-based delivery (127 excerpts)

This theme included statements about the use of phones to deliver the cessation program. Seven subthemes were coded: use of phones compared to in-person care, convenience and cost; interactive features; length of program or messages; literacy issues; privacy issues; and tone of the text messages. In terms of the positive aspects of using phones, participants found that the SMS program compared favorably to in-person care (e.g., “I find these features very good because they create an environment which makes you feel as if you are communicating with a health worker”) (Table 3, Quote 10). There was also considerable enthusiasm for the interactive features—especially the ability to set their own quit date and to get “on demand” support for dealing with cravings, etc. (Table 3, Quote 12). The only suggestion for an additional interactive feature was to provide linkages to further in-person support. While some participants commented positively on the convenience, low cost, and privacy of the SMS program (e.g., “The message coming to my phone is very great. No one has to know about what I’m doing.”), others raised concerns about how to keep the phone charged, network limitations, and literacy issues that may pose challenges to an SMS program (Table 3, Quote 11, 14, 15). Several noted the tone of the messages and appreciated texts, for example, that sounded polite and caring (Table 3, Quote 16). Few excerpts were identified focusing on the length of the program or the text messages, with some participants recommending few texts and others seeing a need for greater program intensity (Table 3, Quote 13).

#### Social support (107 excerpts)

This theme included statements about getting support from others, not wanting to get support from others, or lack of support for quitting at either the individual level or community level. It also included comments about stigma/discrimination, as well as providing support to others for quitting. Most of the excerpts acknowledged the importance of getting support for quitting, and the example text messages indicating that they already had support were found to be inspiring. Respondents also liked the example text messages encouraging them to tell others that they were quitting and to share their successes with others (Table 3, Quote 17). Many participants also mentioned that most of their friends used tobacco, and that it would be important to hang out with non-users rather than tobacco users during quitting (Table 3, Quote 18). There were also several excerpts focusing on support at the community-level, with most of these comments indicating a lack of support for quitting, as well as HIV-related stigma and isolation that would make it difficult to get the support they needed for quitting (Table 3, Quote 19).

#### Dealing with cravings (86 excerpts)

This theme included statements about the importance of focusing on cravings, triggers to tobacco use and barriers to dealing with cravings, suggestions for how to deal with cravings, and liking text messages that were interactive and provided “on demand” support for dealing with cravings (e.g., “If you need support for how to deal with cravings then you should press 4”) (Table 3, Quote 20). In terms of triggers and barriers, participants mentioned other behaviors such as drinking and eating that would increase the likelihood of them using tobacco, lack of support within the community for quitting, poor mental health, and being idle. Suggestions for how to deal with cravings included avoiding “lonely places” where you can use tobacco without others knowing, getting support for dealing with cravings, remembering that health is more important than tobacco, eating something, drinking water, socializing with friends that do not use tobacco and staying away from people who are using tobacco, not carrying tobacco, using determination to avoid cravings, using prayer, and staying busy (Table 3, Quote 21).

#### Getting or staying motivated (85 excerpts)

This theme included statements about what example texts or text content would increase motivation to quit. In terms of the example texts that were presented during the focus groups, many participants provided a general assessment that all of the texts were motivating. Others highlighted as motivating the example texts welcoming them to the program and helping them prepare to quit, encouraging them to get social support for quitting, congratulating them on their progress, checking in on their progress, or focusing on the health benefits of quitting. Other content that participants thought would be motivating included motivation coming from within, acknowledging that quitting is not easy, describing quitting as a gradual process, addressing idleness/lack of employment, and addressing other substance use such as drinking alcohol (Table 3, Quote 22, 23).

#### Benefits of quitting (70 excerpts)

This theme included statements about the positive aspects or outcomes associated with quitting tobacco. Three subthemes were coded: social benefits, financial benefits, and health benefits. **Social benefits of quitting** referred to negative effects of smoking on loved ones who don’t smoke such as secondhand smoke exposure, odor associated with tobacco use, and teaching children that tobacco use is acceptable. In particular, the example text “Quitting will prevent family members from diseases associated with living with a smoker” resonated with several of the participants (Table 3, Quote 26). **Financial benefits of quitting** often referred to spending too much money on tobacco products, sometimes taking priority over other necessities such as food (“So you will go and buy tobacco instead of buying salt which you should use with the rest of the family members and then go beg for salt.”) (Table 3, Quote 24). One participant raised the problem of financial illiteracy “which explains our bad approach on using money in a reckless way.” Several participants pointed out that the example text message asking them to estimate how much they spend on tobacco would be particularly useful to them. Finally, two participants mentioned that one needs to consider that tobacco is a source of income for many users in terms of understanding the financial impact of quitting tobacco. **Health benefits of quitting** largely focused on either the need for more information about the harms of tobacco use/benefits of quitting, or that they already recognized the harms of tobacco use/benefits of quitting. Some of the participants pointed to text messages that they thought were particularly useful in terms of conveying health information, and one thought it was important to convey that the negative health effects of tobacco use are not always visible (Table 3, Quote 25). Two excerpts highlighted the motivating effect of text messages that conveyed that the participant was already improving their health by quitting (i.e. near-term health changes due to quitting). A few excerpts indicated how quitting tobacco use may inspire others to quit (or not start), thereby having a positive effect on others’ health. Finally, two excerpts suggested positive health effects of tobacco use (e.g., improves CD4 count, alleviates pain).

#### Tobacco control interventions at the community level (14 excerpts)

His theme included statements about the need for community-level interventions, existing community-level interventions (in their village or elsewhere), or what a community level intervention might include. One participant noted a need for community-level support (“I think one of the barriers could be things to do with lack of proper supportive structures within the community that one would receive proper guidance on how to deal with cravings”) (Table 3, Quote 27). Two participants mentioned the importance of involving elders in a cessation program (e.g., “… they should be in best position to explain how to reduce and eventually leave tobacco use”) and four suggested making the program available to everyone in the community because “almost all people here use tobacco,” such as through radio or a day-long program focused on educating the community about the effects of tobacco (Table 3, Quote 28). Several participants suggested banning tobacco sales or discouraging people in the community from growing tobacco, but others noted a need to support or provide another livelihood to those who stopped growing tobacco. Finally, one participant suggested involving PLWH who quit tobacco as role models in the community who could then help others quit.

*Tobacco source and income (9 excerpts).* This theme included statements about where they purchased or otherwise obtained tobacco, as well as statements about the cultivation of tobacco and income associated with cultivation. One participant mentioned how elders are a reliable source of free tobacco (Table 3, Quote 29). Another indicated that people will give you tobacco, whether you want it or not, because they already know you are a tobacco user. There were also suggestions for reducing access to tobacco, such as discouraging farmers from growing tobacco and arresting those who import tobacco into the country (Table 3, Quote 30). Several participants discussed how tobacco cultivation is an important source of income for many in their community and that needs to be considered when implementing a tobacco cessation program.

### HSW focus group qualitative results

Ten subthemes emerged for ***barriers healthcare providers encounter when helping PWLH quit tobacco***. In terms of PLWH-level barriers, many participants noted the denial of tobacco use among clients as being a barrier to helping PLWH quit tobacco (Table 4, Quote 1). There were also apparent knowledge gaps about tobacco cessation, with respondents noting that many people lack knowledge on the importance of quitting (Table 4, Quote 2). Addiction and the use of other substances such as alcohol were also mentioned frequently (Table 4, Quote 3,4). Social network-level barriers included peer influences and a lack of family support, detailing that PLWH will smoke in groups and do not have family support in quitting (Table 4, Quote 5.6). For healthcare providers themselves, knowledge gaps in delivering tobacco cessation advice was mentioned in addition to tobacco cessation not being a priority due to clinics being overwhelmed (Table 4, Quote 7,8). At the community-level there was a need to better understand community perspectives through sensitization and research to inform strategies for helping clients quit tobacco use. Tobacco was also mentioned as being a cheap and easily available source of income for clients, with health service workers hoping to address this through interventions (Table 4, Quote 9,10).

**Table 4:** Health services worker focus group themes and example quotes

Theme		Subthemes and Example Quotes
Barriers health care providers encounter when helping PLWH quit tobacco	PLWH-level barriers	**Denial of tobacco use:** *“One big issue is denial. In your presence they can deny that they smoke but if they go back they continue smoking (1).”* **Knowledge gap about tobacco cessation: *“****To me, I think these people lack knowledge about the risks and dangers of smoking and the importance of quitting smoking because if they knew, they would not continue doing that (2).”* **Addiction to using tobacco:** *“According to my observation, those clients believe that they are addicted and they cannot leave tobacco and yet that is just a mindset. So, I think this can be addressed by continuous counselling (3).”* **Use of other substances:** *“My assumption is that these….these clients of ours….aaaa….may not only have an addiction towards smoking, but may also have….they may also have other vices that they are actually dealing with, be it alcohol whatever else it is (4).”*
	Social network-level barriers	**Peer influences:** *“Yes, many of them wish to quit but peer influence is still a challenge. They stop for few days but when they get in groups, they start smoking again (5).”* **Lack of family support:** *“The challenge is lack of family support. We can counsel and give them the necessary support as health workers but when they go home, there is no one to support them quit smoking (6).”*
	Health care provider-level barriers	**Knowledge gap in tobacco cessation:** *“Most of us have never had any form of training on tobacco use. So, sometimes it is hard to give the right information to the client. So, I think all these health workers should be empowered with knowledge about tobacco use (7).”* **Helping clients quit tobacco is not a priority:** *“I think we need priority topics to talk and discuss with our clients because at times we take a lot of time talking about adherence and other things but when it comes to tobacco use, we don’t give enough time. This could be because we don’t see tobacco use as a priority (8).”*
	Community-level barriers	**Need to better understand community perspective to inform strategies for helping clients quit tobacco:** *“Since these issues originate from the community, we need to do a lot of sensitization so that we understand the root cause from the community perspective in order to get strategies on what to do (9).”* **Tobacco is cheap, easily available, and a source of income: *“****I don’t know if this has been put into consideration, when you started the program, the source of the tobacco, is there any plans on the restriction on the affirmation of the tobacco, like because here is someone who actually sells tobacco, they smoke it, they plant it and they sell. So for me I feel it will be hard for this person to, you tell them to stop but that’s their way of living. Someone plants it year in, year out, I don’t know if there are restrictions that can be put in place in terms of planting tobacco in Zambia (10).”*
Use of SMS as a program to help PLWH quit tobacco		**Challenges in how to use phone, keep phone secured and charged, and network issues:** *“To me, it may not work since most of them don’t have phones. Besides, network is a problem in most rural areas here. Yes, illiteracy. Many of them may not know how to use a phone, I mean how to check the SMS or even call. So, it may get damaged in the process (11).”* **SMS may not work due to literacy issues and language barriers:** *“To me SMS may not work because the majority of these people have not gone to school and cannot read English and others cannot read the local language too. So, this may not be effective (12).”* **Getting a phone will motivate clients to participate:** *“This will now motivate them to participate in this program because they will know that at the end the phone will remain with them. So, this should be made clear to them that the benefit will be the phone (13).”* **SMS will keep clients engaged in quitting:** *“I think the SMS will be a good strategy because it will constantly remind the tobacco users to not use the….to not smoke or to not use tobacco. It’s a good encouragement and will always encourage them to stop using it and….yeah, basically it will remind them and will also be an encouragement (14).”*
Critical elements in tobacco cessation services for PLWH		**Increase awareness of dangers of tobacco use through community activities, radio advertising, posters, social media, etc.:** *“There I would say, there should be ongoing adverts on radios talking about the dangers of tobacco so that these messages can reach everybody, many people listen to radios and then phone SMS.” “Putting posters with photos showing the dangers and effects of smoking besides using the SMS, maybe by seeing it physically, it will scare them and help them quit tobacco (15).”* **Involve health care providers to review client’s progress and counsel them on a regular basis: *“****For me, for these clients to stop, we need to continue counselling and talking to them (16).”* **Involve treatment supporters because they know the clients better:** *“These treatment supporters are important because they are the ones who stay with these clients in the hospital or at home. They know the clients very well and are very well conversant with what the clients do on a daily basis…. So, it will be good to bring them onboard so that they should be able to know what they should tell these clients and what we expect from them so that when the client does something opposite, the supporter will be able to guide or correct them or help them along that line (17).”* **Conduct group meetings of participants to share their experiences:** *“I was thinking that maybe we can have some support groups, whereby they encourage each other in the presence of the providers (18).”* **Provide participants with alternative activities to tobacco use:** *“I think it is failure to not offer an alternative. You might tell someone to quit but what will they do? Because others lack recreational facilities, so it’s difficult to tell them to quit, unless you have the same replacement therapy. That’s when it will be easy for us to convince them because we will tell them that instead, this (19).”* **Provide participants with pharmacotherapy:** *“I have seen some tablets which are used to reduce the craving for the tobacco. We have those available so that even as we are educating our clients, tell them about the treatment and we do not just tell them about the treatment but also provide the treatment itself (20).”* **Provide participants with information about quitting:** *“So what’s important is to make available the information to the people, explain to them, even the bad part of continuous smoking, the side effects and things like that and when they appreciate that, they can become ambassadors….they can become even ambassadors to other people to say that okay, it’s necessary to quit, yeah (21).”* **Importance of confidentiality and no stigma for program participation:** *“First, we need to give them assurance that what they are going to tell us is going to be confidential, this will help them open up (22).”*

Bold values indicate subthemes identified within main themes

Five subthemes emerged for ***the use of SMS as a program to help PLWH quit tobacco use*.** Health service workers discussed potential drawbacks of using SMS, mentioning challenges in how to use the phones, keeping phones secure and charged, network issues, literacy issues, and language barriers (Table 4, Quote 11,12). SMS was also discussed as being a motivating factor as getting a phone could motivate PLWH to participate and SMS can potentially keep PLWH engaged in quitting (Table 4, Quote 13). Respondents also encouraged involving expert clients and village health teams (VHTs) in the cessation process (Table 4, Quote 14).

Finally, eight subthemes emerged for ***critical elements in tobacco cessation services for PLWH***. The most dominant subtheme was to increase general awareness of the dangers of tobacco use through community activities, radio advertising, posters, social media, and so forth (Table 4, Quote 15). Participants discussed the importance of providing PLWH social support for quitting: involving health care providers in reviewing clients’ progress and counseling them regularly; involving treatment providers who know the client in the quitting process; and offering group meetings of clients to share their experiences (Table 4, Quote 16,17,18). Other topics mentioned included providing other resources to PLWH for quitting: alternative activities to tobacco use, pharmacotherapy for quitting, and information on quitting (Table 4, Quote 19,20,21). Participants also emphasized the importance of confidentiality and no stigma for program participation (Table 4, Quote 22).

### PLWH usability testing survey results

#### Preferences for TMI delivery

As shown in Table 1, on average, the usability testing participants wanted to receive 4 to 6 text messages per day. Similar to results from the PLWH focus group survey, the number varied somewhat depending on the stage of the quit attempt. In terms of when to receive texts, the usability testing sample did not have a clear preference for time of day.

#### Feasibility and acceptability of TMI

As shown in Table 1, usability testing participants rated the SMS program as being “very” to “extremely” helpful if they were trying to quit tobacco and relevant in terms of text message content. Further, they indicated that it was “very” to “extremely” likely that they would use a program like this one if they were trying to quit tobacco. In terms of problems experienced using the TMI for 3 weeks on their own phone, 36% reported trouble keeping their phone charged, 21% reported their phone was lost, 21% reported their phone was stolen, 14% reported feeling uncomfortable when others saw comments on their phone, and 14% reported feeling uncomfortable when others saw text messages about tobacco cessation on their phone.

## Discussion

This paper describes results from the formative phase of a larger study that involves conducting the first large-scale RCT to test the efficacy of both a tailored TMI for tobacco use cessation and nicotine replacement therapy among PLWH in Uganda and Zambia. Through focus group discussions and usability testing, PLWH offered insight into major barriers to tobacco cessation and agreed that implementing the targeted messaging program would provide them with the knowledge and motivation needed to quit.

This qualitative work highlighted the need to adapt the existing evidence-based TMI to ensure cultural and contextual relevance for PLWH in SSA. Adaptations focused largely on effectively designing messages that target both combustible and noncombustible tobacco use and acknowledge the unique challenges of tobacco cessation, including limited knowledge, community perceptions, and cultural practices. Based on focus group feedback, we expanded the text message library to include messages addressing the use of tobacco for easing hunger, enhancing alertness, and conforming to community norms that view tobacco as being socially acceptable. Given that PLWH expressed a preference for messages that provide education on the negative health effects of tobacco use and how to quit, we also added more content on how tobacco use affects the body (with a particular focus on health issues facing PLWH) and practical tips for dealing with cravings while quitting. Previously adapted TMI studies in LMICs have included similar messages, with participants requesting loss-framed messages that emphasize health risks over the benefits of quitting [30].

Both PLWH and HSW expressed support for adopting the TMI into HIV clinics, reinforcing its perceived acceptability and feasibility within existing healthcare structures. Usability testing among PLWH specifically highlighted the attractiveness of the interactive features and the inspiring tone of the intervention. Similar text programs that have included interactive features like setting a quit date and tracking tobacco use have also seen positive responses from users [31]. Many studies have shown that the interactivity and personalization of TMI features enhances overall user appeal and increases the likelihood of continued participation [32, 33].

Feedback on the TMI delivery was also critical in terms of informing more practical implementation decisions, including whether the provision of phones and chargers was needed for the RCT, the parameters of TMI delivery that are important for this population (e.g., number and timing of texts), and language preferences. Given that reviews of technology-delivered interventions in LMICs have highlighted challenges such as digital literacy, technology accessibility, and cost implications, participants were asked to discuss these potential barriers and their likelihood to continue use of the program [34]. Upon completion of usability testing, we acknowledged the need to purchase solar chargers for all participants and made messages available in both English and the local language. In terms of security and confidentiality issues, most participants noted that phone loss and privacy were not areas of major concern for them, which is consistent with a recent TMI reminder intervention in SSA [35]. Overall, study participants indicated that the adapted cell phone-delivered TMI holds considerable promise for tobacco cessation for PLWH in Uganda and Zambia.

Findings from this formative work should be considered in light of the study’s limitations and strengths. In terms of limitations, usability testing participants were asked to use their own phones, rather than being provided with phones and chargers. Further, they were asked to use the TMI for three weeks, rather than the full six weeks of the actual protocol length. Both of these factors may have influenced the feedback that they provided on the TMI’s acceptability and feasibility. Notable strengths of this formative work include soliciting feedback from multiple stakeholders—namely, PLWH interested in quitting tobacco and health services workers from the HIV clinics where these PLWH seek services—to inform the content and delivery of the Quit4Life + intervention. Another key strength is that the formative work was conducted in both Uganda (two regions) and Zambia (two regions) where Quit4Life + will be evaluated through a randomized controlled trial, as these two countries differ in terms of tobacco use patterns, policy environments, and health care resources.

## Conclusion

Tobacco use remains a significant public health challenge among PLWH in SSA, yet effective, scalable interventions targeting this population are lacking. Findings from the formative phase of this project—including focus group discussions and usability testing—guided key adaptations to the Quit4Life + text message-based tobacco cessation intervention for PLWH in Uganda and Zambia, ensuring cultural relevance, acceptability, and feasibility. Participants found the interactive intervention informative and supportive, highlighting key barriers to quitting tobacco for PLWH and challenges in providing cessation support by healthcare workers within clinics. These findings support the potential effectiveness of the text-based program and offer valuable insights for its evaluation in Uganda, Zambia, and beyond.

## Data Availability

Unidentified data from this study will be available from the corresponding author on reasonable request one year after all aims of the project are completed. Requestors of data will be asked to complete a data-sharing agreement that provides for (1) a commitment to using the data only for research purposes and not to identify any individual participant; (2) a commitment to securing the data using appropriate computer technology; and (3) a commitment to destroying or returning the data after analyses are completed.

## Appendix 1. Sample text messages that were presented to focus group participants

**Table:**

You are welcome to join this program intended to support tobacco users to quit It is a free SMS program supported by Ministry of Health, Makerere University School of Public Health & Centre for Tobacco Control in Africa Quitting works best when you are prepared. Stay with us & you won’t have to rely on willpower alone. We will support you how to quit
Well done! You have made a lifesaving decision to quit tobacco use Start reducing how much tobacco you use in a day Identify ways of dealing with craving Tell a friend! Try quitting with someone. You will have a friend to talk to about how you feel and this support will help keep you on track
Quitting will improve your health Quitting will prevent family members from diseases associated with living with a smoker Quitting will prevent you from getting tobacco use related diseases Estimate the amount you spend on tobacco products every day in a year, it could be a lot
Set a quit date and tell it to your support person
Get more support whenever you need it! Type 3 for emotional support, 4 for advice to overcome cravings, or 9 for information on why to quit Cravings are real. They won’t go away immediately, but feeding them only makes them stronger. What is your craving level? Reply: HI, MED, or LOW We know quitting is tough but stay strong! We all have bad days, and you will get through this. Do whatever to boost your mood-just do not smoke
Today is your QUIT date! It is here & you can do this! You are stronger than you think, so be positive. Are you ready? Respond with READY or NOT ● READY: Great–let’s get started! Have mints, water, and snacks to get you through the day. Life is all about choices and you will not regret this one ● NOT: When you are ready to pick a new date, we hope you sign up again by texting READY. You can even sign up again today 24 h quit: Tell your support person and do something nice for yourself
*7 days cessation:* Celebrate your success
*4 weeks cessation:* You’ve done it! Share the good news with friends!
Great job! Keep staying strong and tobacco free. We know it is not easy, but it is totally worth it
How are you doing? Are you still tobacco free? Reply: YES or NO

## Appendix 2. Consolidated criteria for reporting qualitative research (COREQ): 32-item checklist

**Table:**

Domain 1: Research team and reflexivity
Personal Characteristics
1. Interviewer/Facilitator—interviews were conducted by two trained study research assitants
2. Credentials—researchers credentials were MPH
3. Occupation—at the time of the study interviewer occupations were research assistants
4. Gender—Both researchers were male
5. Experience and training—both researchers had previous experience in conducting focus group discussions and were trained as research assistants for the study
6. Relationship established—interviewers introduced themselves prior to focus group discussions
7. Participant knowledge of the interviewer—before focus group discussions, participants were given background of the study purpose based on a standard interview guide
8. Interviewer characteristics—interviewers noted no bias or conflict of interest in facilitating the focus group discussions
Domain 2: Study design
Theoretical framework
9. Methodological orientation and theory –
Participant selection
10. Sampling—Participants were selected by expert clients at selected clinics
11. Method of approach—Participants were approached face-to-face for participation
12. Sample size—There were 50 participants across all focus groups
13. Non-participation—no participants approached refused to participate in the study
14. Setting of data collection—Data was collected in participating HIV clinics
15. Presence of non-participants –Other than the participants and researchers, no one was present
16. Description of sample—The study sample consisted of people living with HIV and health service workers being treated or working at the participating clinics
Data collection
17. Interview guide—an interview guide was used to facilitate focus group discussions. It was not pilot-tested before use
18. Repeat interviews—There were no repeat interviews conducted
19. Audio/visual recording—Interviews were audio recorded
20. Field notes—Field notes were made before and after the interview
21. Duration—The duration of focus group discussions was one hour
22. Data saturation—Data saturation was discussed and monitored throughout focus group discussions
23. Transcripts returned—Transcripts were not returned to participants for comments or corrections
Domain 3: Analysis and findings
24. Number of data coders—There were 3 total data coders
25. Description of coding tree—Authors provided key themes developed using the standard discussion guides
26. Deviation of themes—Themes were derived from the data
27. Software—Dedoose software was used to manage the data
28. Reporting—Participant checking—Participants did not provide feedback on the findings
29. Quotations presented—Participant quotations were presented to illustrate themes. Quotations were not identified
30. Data findings and consistency—There is consistency between the data presented and the findings
31. Clarity of major themes—Major themes were presented clearly in the findings
32. Clarity of minor themes—There is discussion and descriptions of minor themes

## Appendix 3. Standard discussion guide for people living with HIV focus groups

*INSTRUCTIONS*: Use this guide only for HIV patients at the facility.

ELIGIBILITY CRITERIA for patients at the facilities. Participants must be HIV positive and have used any type of tobacco product in the past 30 days. The particpants may be male or female.

*Introductions**: *Thank you for coming today. We are from Makerere University Scool of Public Healh working with University of Southern California and RAND Corporation in America. We would like to get your views regarding a program to help HIV positive people quit tobacco use. Before we begin, let me read a consent form that describes the purpose of the study, the benefits and risks as we as possibility hey withdraw at whichever the stage of the interview. I will then ask whether you agree to participate in the focus group.

READ CONSENT FORM and obtain consent from all participants before you commence with the group discussion.

Please introduce yourself and tell us a little bit about yourself.

As I just mentioned during the informed consent process, we would like to get your feedback on a SMS program to help PLWH quit tobacco called Text2Quit. The goal of this program is to help patients like you quit tobacco use and to stay tobacco-free. Patients who use the program will be asked to set a quit date. Once they sign up for the program, they will be provided with a cell phone and then will start receiving text messages that will provide ongoing support throughout the quitting process. Messages will be sent in the days leading up to their quit date, on the actual day they quit, and then for 3 months after their quit date.

The program that we’re considering using is an SMS program called Text2Quit:

Now I’m going to share with you some examples of text messages about quitting tobacco so you can get an idea of what the program will look like.

The first set of texts are ones that you would receive on the *days before your quit day*. These messages are designed to help you prepare to quit.

[Show “pre-quit” texts].

1. Imagine that you are preparing to quit tobacco tomorrow or the next day.
  - What do you think about the content of these texts—how useful would they be to you?
  - What do you think about the interactive features, such as being able to text your main reasons for wanting to quit and getting information on using nicotine replacement?
  - What other messages or links to other information could we send that would be useful as you prepare to quit?

The next set of messages are ones that you would receive on *your actual quit day*. These messages are designed to get you through this key day, when you’re trying to avoid tobacco (and maybe avoid situations or people who remind you of tobacco). You’re also probably starting to feel some nicotine cravings.

[Show “quit day” texts].

2. Imagine that today is your quit day.
  - What do you think about the content of these texts—how useful would they be to you?
  - What do you think about the interactive features, such as the link providing more information on quitting?
  - What other messages or links to other information could we send that would be useful to get you through your quit day?

The next set of messages are ones you would receive on the *day after your quit day*. On this day you’re probably dealing with nicotine cravings—maybe you’re distracted or moody because of the cravings. You’re trying to stay tobacco-free, but maybe you’ve slipped and used tobacco. Or at least thought about it. Maybe your friends are using tobacco around you and you’re in deedssome support for quitting.

[Show “post-quit day” texts].

3. Imagine that you quit tobacco yesterday and you’re dealing with some of the issues I just mentioned.
  - What do you think about the content of these texts—how useful would they be to you?
  - What do you think about the interactive features, such as being able to get more support if you have a craving or you slip and use tobacco?
  - What other messages or links to other information could we send that would be useful the day or two after quitting?

After you get over the “hump” of quit day, we would keep sending you text messages for the next 3 months to provide ongoing support for staying tobacco-free. These texts would focus on issues such as:

- Dealing with cravings
- Staying motivated to quit
- Getting social support for quitting
- Beneficial outcomes associated with quitting

4. Let’s go through some of these issues. The first one is dealing with cravings to tobacco use. What are some specific barriers to dealing with cravings and temptations these we need to address with this program? Do you have some examples of texts that you would find particularly helpful?
  - [write down suggestions… *go through this process with other program components*]
5. What’s missing from this list in terms of issues that we should be addressing to help tobacco users like you quit?
6. Are there certain issues that you think are more important than others and should be focused on more with the text messages?
7. If you were trying to quit tobacco and found yourself in a situation where you were really tempted to use it, what would be most helpful in terms of getting you through the situation and staying tobacco-free?
8. In your opinion? What’s missing from this program that you think would be useful to help tobacco users like yourself quit?
9. What other suggestions do you have for helping us improve this program?

Thank you so much. Next we are going to ask you to complete a brief questionnaire.

## Appendix 4. Standard discussion guide for health service workers focus groups

*INSTRUCTIONS*: Use this guide only for health service workers at the facility.

ELIGIBILITY CRITERIA for heath workers working at the facilities. Participants must have some knowledge of HIV and tobacco use, if possible.

Introductions: Thank you for coming today. We are from Makerere University Scool of Public Healh working with University of Southern California and RAND Corporation in America. We would like to get your views regarding how to help people who are HIV positive to quit using tobacco. Before we begin, let me read a consent form where the purpose, objectives, risks and benefits will be explained. I will then ask whether you agree to participate in the focus group.

READ CONSENT FORM and obtain consent from all participants before you commence with the group discussion.

Let us go around the room: please introduce yourself and tell us a little bit about yourself. [MAKE NOTES ON EACH PERSON’S GENDER, AGE, MAIN OCCUPATION, MARITAL/RELATIONSHIP STATUS, CHILDREN].

Introduction

1. How is the situation of HIV positive clients who smoke and receive their services at this clinic. Probe for degree of severity compared to those that do not smoke.
2. Would you say that some HIV positive clients wish to quit smoking? Explain your response.

Barriers

1. What do you think are some of the major barriers that health care providers might encounter in helping PLWH quit using tobacco? What are some strategies for addressing these barriers?
2. Do you have an opinion reagarding the use of SMS as a program to help PLWA quit use of tobacco.
3. Are there any additional barriers that would be specific to using an SMS program to help PLWH quit tobacco? What are some strategies for addressing these barriers?
4. What are some of the other issues that we should be aware of in helping health care providers to implement a tobacco cessation program for PLWH?

Accelerators

1. What do you see as the most critical elements in tobacco cessation services for PLWH? (What needs to be done to help PLWH quit tobacco use?).
2. What do you think would be the most effective tobacco cessation program to use with PLWH? What are the core or critical elements of this program?
3. What changes—or adaptations—would need to be made to an existing program to make it as effective as possible for PLWH, if anything?
4. What can we do in order to encourage PLWH to participate in a tobacco cessation program?
5. Any other comments?

Thank you so much.

## Abbreviations

AIDS: Acquired immunodeficiency syndrome
ART: Anti-retroviral therapy
CTCA: Center for Tobacco Control Africa
HIV: Human immunodeficiency virus
NRT: Nicotine replacement therapy
PLWH: People living with HIV
SMS: Short message/messaging service
SSA: Sub-Saharan Africa
VHT: Village health team
